# Supplementary material for: Synergism of Carbamoylated Erythropoietin and Insulin-like Growth Factor-1 in Immediate Early Gene Expression
Source: Life (Basel). 2023 Aug 29;13(9):1826. doi: 10.3390/life13091826 (PMC10532867; doi:10.3390/life13091826)
Supplement: Supplementary file 1 [file life-13-01826-s001.zip › Supplementary Figure Captions.pdf]

Supplementary Figure Captions

Figure S1. Trophic factor-induced phosphorylation of ERK and AKT in PC-12 cells. Western blot of PC-12 cells treated with CEPO (100 ng/mL), IGF-1 (100 ng/mL), and CEPO + IGF-1 (50 ng/mL each) for (**A**) 3 hours or (**D**) 5 hours. (**B, C**) Quantification of western blot bands from (A) (N=3). (**E, F**) Quantification of western blot bands from (D) (N=3). Error bars are ± SD. Significance was determined when compared to the vehicle-treated group. *$p < 0.05$, **$p < 0.01$, ***$p < 0.001$, ****$p < 0.0001$ one-way ANOVA with Dunnett's multiple comparisons post hoc test.

Figure S2. Hippocampal regions examined in the immunohistochemistry experiments. (**A**) DAPI-stained rat hippocampal section at Bregma = -3.30 mm. White boxes labeled 1 and 2 represent the two regions of interest used during IHC analysis. (**B**) Schematic of rat hippocampus at Bregma = -3.36 mm to show regions of interest. (**C**) Representative image from CA3 region, corresponding to box 2 from (A). (**D**) Representative image from CA1 region, corresponding to box 1 from (A). DG = dentate gyrus.

Table S1. Genes used during qPCR and dPCR analysis with corresponding forward and reverse primers sequences.
