# Supplementary material for: Synergism of Carbamoylated Erythropoietin and Insulin-like Growth Factor-1 in Immediate Early Gene Expression
Source: Life (Basel). 2023 Aug 29;13(9):1826. doi: 10.3390/life13091826 (PMC10532867; doi:10.3390/life13091826)
Supplement: Supplementary file 1 [file life-13-01826-s001.zip › Supplementary Figures_merged.pdf]

Figure S1.

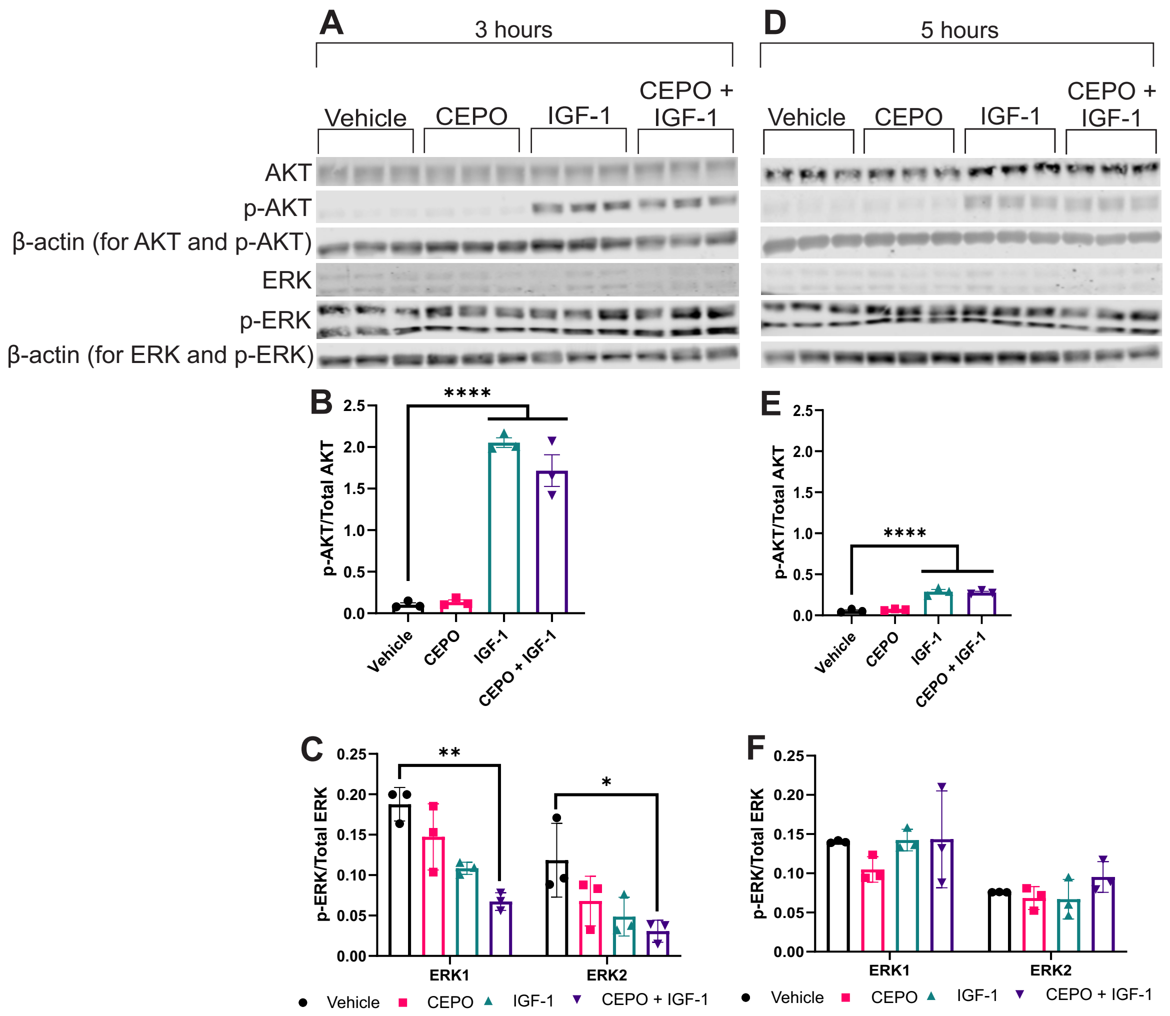

A
3 hours
Vehicle
CEPO
IGF-1
CEPO + IGF-1
AKT
p-AKT
β-actin (for AKT and p-AKT)
ERK
p-ERK
β-actin (for ERK and p-ERK)
D
5 hours
B
p-AKT/Total AKT
****
C
p-ERK/Total ERK
**
*
ERK1
ERK2
E
F
Vehicle CEPO IGF-1 CEPO + IGF-1

Figure S2.

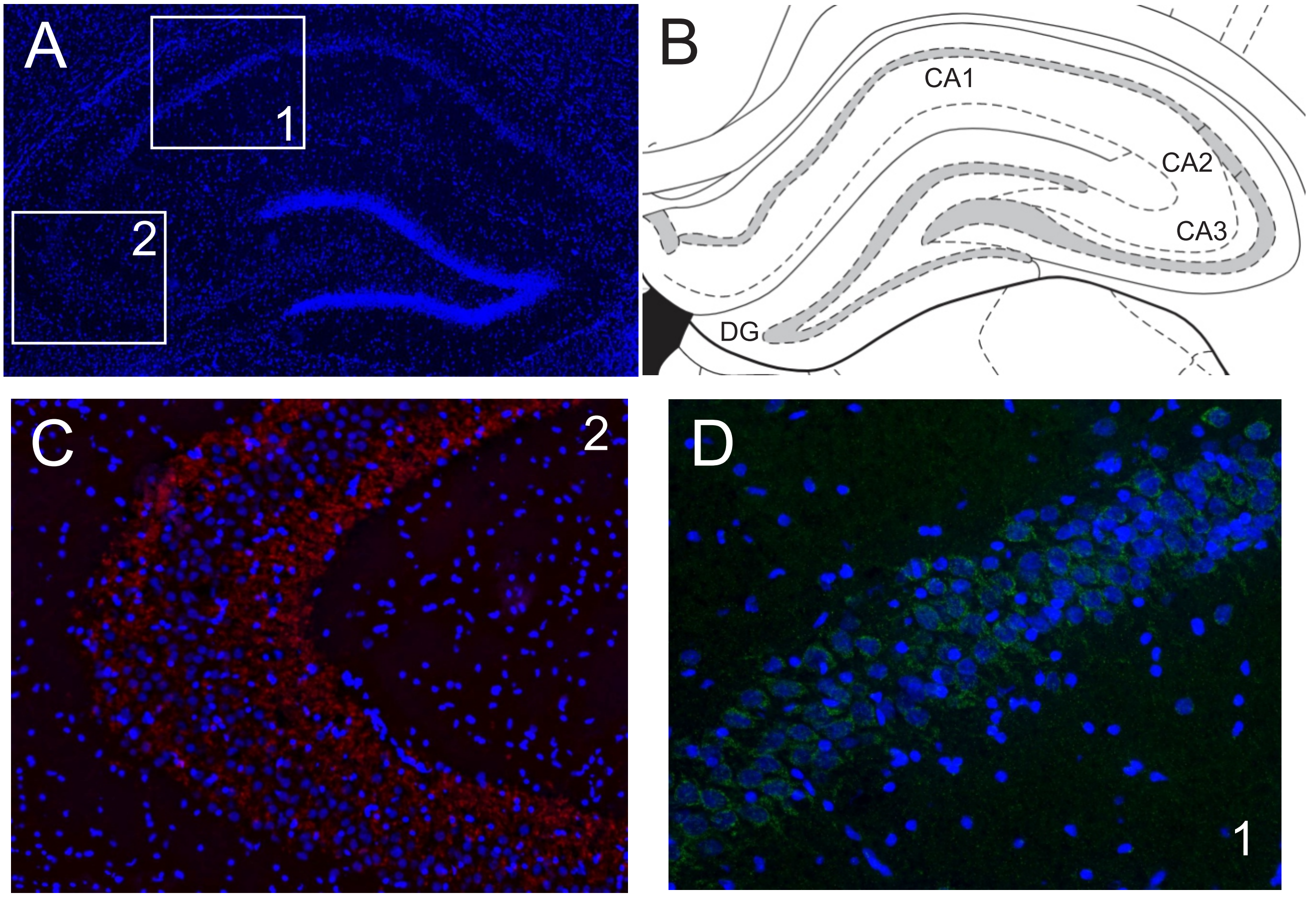

Hippocampus (CA3)

Hippocampus (CA1)

Table S1.

Gene	Gene Name	NCBI Reference Sequence	Forward	Reverse
CYC (PPIA)	Cyclophilin A (peptidylprolyl isomerase A)	NM_017101.1	caggtcctggcatcttgtcca	tgcttgccatccagccactc
cFos	Fos proto-oncogene	NM_022197.2	gcgcagatctgtccgtctct	ccacggaggagaccagagtg
FosB	FosB proto-oncogene	NM_001256509.1	gcctggagtttgtcctggtg	agccgaagccgtcttcctta
JunB	JunB proto-oncogene	NM_021836.2	caagtactgccggcctccta	cagaaggcgtgtcccttgac
Egr1	Early growth response 1	NM_012551.3	ctgcctaccctgccaccaag	gctgctgggtacggttctcc
Npas4	Neuronal PAS domain protein 4	NM_153626.1	ctcctcccagcacagcattc	atccctcctgggcgaagtaa
Inhba	Inhibin subunit beta A	NM_017128.2	tgctcctgggcaagaagaag	agcctgcagcatgaggaaag
ARC	Activity-regulated cytoskeleton-associated protein	NM_019361.2	agcgggacctgtaccagaca	cgcagaaagcgcttgaactt
tPA	Tissue-type plasminogen activator	NM_013151.3	gtgtgcccaggagagcagtt	ctccttcagccggtcagaga
Nptx2	Neuronal pentraxin-2 precursor	NM_001034199.1	ggcagatttgatgccacaca	tgtttccaggcatgttcgtg
BDNF	Brain-derived neurotrophic factor	NM_001270630.1	gctgcgcccatgaaagaagc	agacctctcgaacctgccct
CD131	Colony stimulating factor 2 receptor subunit beta	NM_133555.1	aggtctcctcagcccaaagc	tggaggcagctccacgtaat
EPOR	Erythropoietin receptor	NM_017002.2	aaagggtggaggtcctggaa	tccagaatccgctgaagctc
IGF1R	Insulin-like growth factor 1 receptor	NM_052807.2	gagctggagatggagctgga	ccgttctcagccttgtgtcc
IR (Insr)	Insulin receptor	NM_017071.2	tggcatggcatacttgaacg	ttcagggactcgggtgacat

Supplementary Figure Captions

Figure S1. Trophic factor-induced phosphorylation of ERK and AKT in PC-12 cells. Western blot of PC-12 cells treated with CEPO (100 ng/mL), IGF-1 (100 ng/mL), and CEPO + IGF-1 (50 ng/mL each) for (**A**) 3 hours or (**D**) 5 hours. (**B, C**) Quantification of western blot bands from (A) (N=3). (**E, F**) Quantification of western blot bands from (D) (N=3). Error bars are ± SD. Significance was determined when compared to the vehicle-treated group. *$p < 0.05$, **$p < 0.01$, ***$p < 0.001$, ****$p < 0.0001$ one-way ANOVA with Dunnett's multiple comparisons post hoc test.

Figure S2. Hippocampal regions examined in the immunohistochemistry experiments. (**A**) DAPI-stained rat hippocampal section at Bregma = -3.30 mm. White boxes labeled 1 and 2 represent the two regions of interest used during IHC analysis. (**B**) Schematic of rat hippocampus at Bregma = -3.36 mm to show regions of interest. (**C**) Representative image from CA3 region, corresponding to box 2 from (A). (**D**) Representative image from CA1 region, corresponding to box 1 from (A). DG = dentate gyrus.

Table S1. Genes used during qPCR and dPCR analysis with corresponding forward and reverse primers sequences.
