# Supplementary material for: Synergism of Carbamoylated Erythropoietin and Insulin-like Growth Factor-1 in Immediate Early Gene Expression
Source: Life (Basel). 2023 Aug 29;13(9):1826. doi: 10.3390/life13091826 (PMC10532867; doi:10.3390/life13091826)
Supplement: Supplementary file 1 [file life-13-01826-s001.zip › Table S1.pdf]

Gene	Gene Name	NCBI Reference Sequence	Forward	Reverse
CYC (PPIA)	Cyclophilin A (peptidylprolyl isomerase A)	NM_017101.1	caggtcctggcatcttgtcca	tgcttgccatccagccactc
cFos	Fos proto-oncogene	NM_022197.2	gcgcagatctgtccgtctct	ccacggaggagaccagagtg
FosB	FosB proto-oncogene	NM_001256509.1	gcctggagtttgtcctggtg	agccgaagccgtcttcctta
JunB	JunB proto-oncogene	NM_021836.2	caagtactgccggcctccta	cagaaggcgtgtcccttgac
Egr1	Early growth response 1	NM_012551.3	ctgcctaccctgccaccaag	gctgctgggtacggttctcc
Npas4	Neuronal PAS domain protein 4	NM_153626.1	ctcctcccagcacagcattc	atccctcctgggcgaagtaa
Inhba	Inhibin subunit beta A	NM_017128.2	tgctcctgggcaagaagaag	agcctgcagcatgaggaaag
ARC	Activity-regulated cytoskeleton-associated protein	NM_019361.2	agcgggacctgtaccagaca	cgcagaaagcgcttgaactt
tPA	Tissue-type plasminogen activator	NM_013151.3	gtgtgcccaggagagcagtt	ctccttcagccggtcagaga
Nptx2	Neuronal pentraxin-2 precursor	NM_001034199.1	ggcagatttgatgccacaca	tgtttccaggcatgttcgtg
BDNF	Brain-derived neurotrophic factor	NM_001270630.1	gctgcgcccatgaaagaagc	agacctctcgaacctgccct
CD131	Colony stimulating factor 2 receptor subunit beta	NM_133555.1	aggtctcctcagcccaaagc	tggaggcagctccacgtaat
EPOR	Erythropoietin receptor	NM_017002.2	aaagggtggaggtcctggaa	tccagaatccgctgaagctc
IGF1R	Insulin-like growth factor 1 receptor	NM_052807.2	gagctggagatggagctgga	ccgttctcagccttgtgtcc
IR (Insr)	Insulin receptor	NM_017071.2	tggcatggcatacttgaacg	ttcagggactcgggtgacat
