# Supplementary figures and images for: Synergism of Carbamoylated Erythropoietin and Insulin-like Growth Factor-1 in Immediate Early Gene Expression
Source: Life (Basel). 2023 Aug 29;13(9):1826. doi: 10.3390/life13091826 (PMC10532867; doi:10.3390/life13091826)

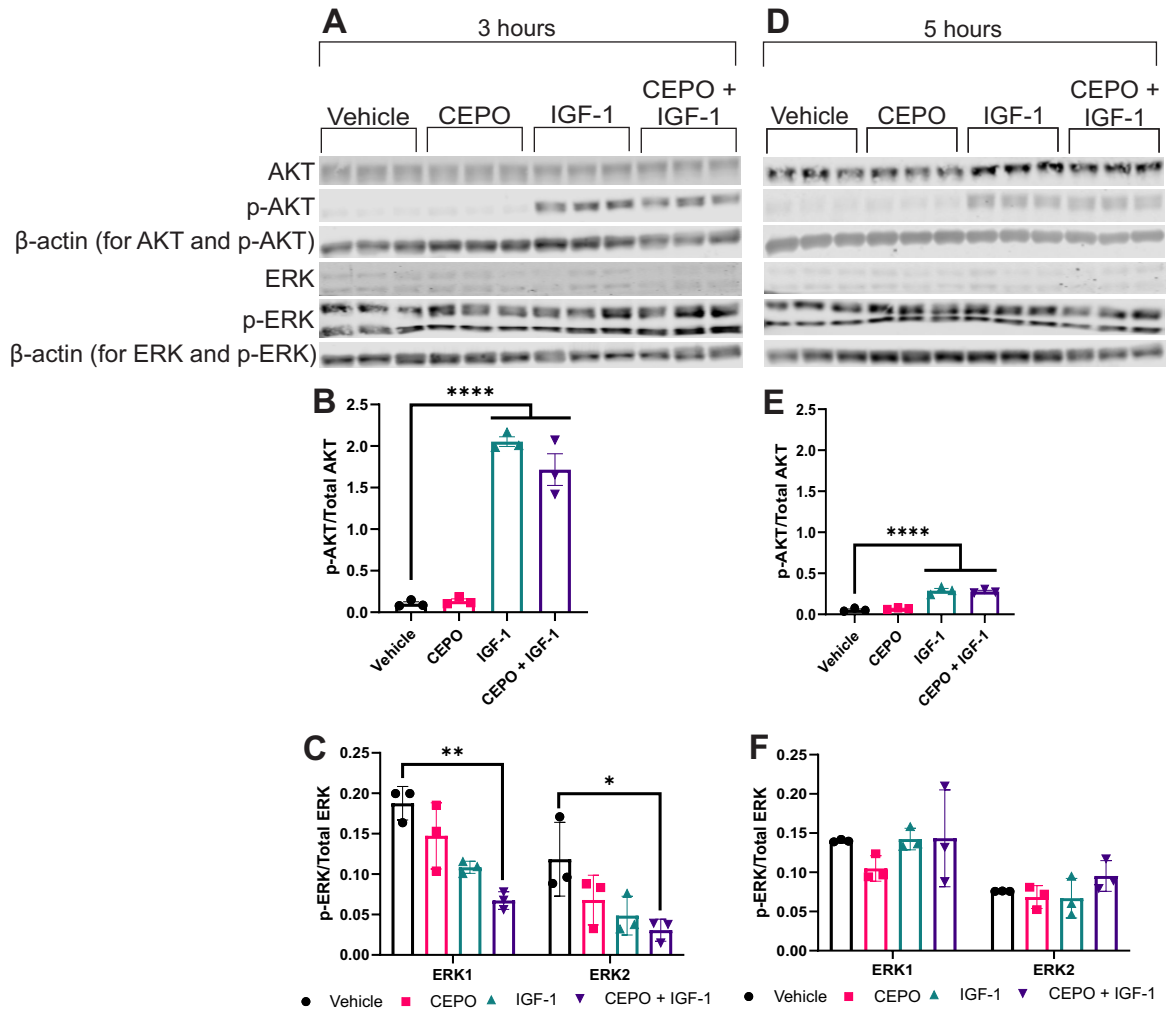

Supplement: Supplementary file 1 [file life-13-01826-s001.zip › Figure S1 - Western Blot (3&5h).pdf]

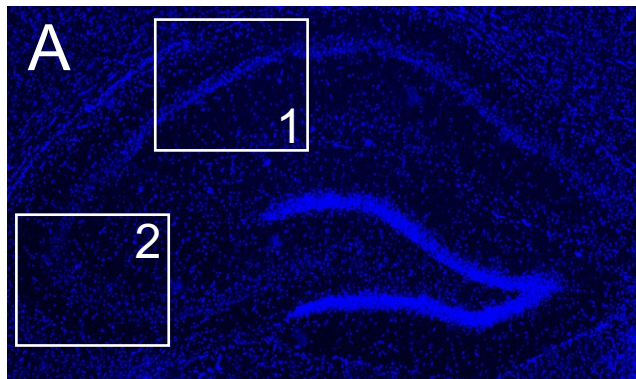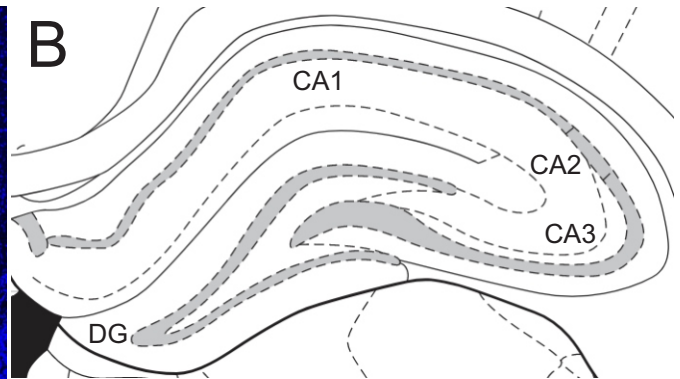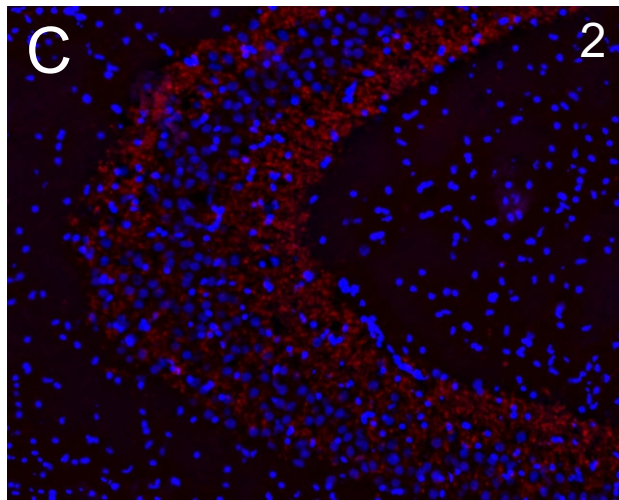

Hippocampus (CA3)

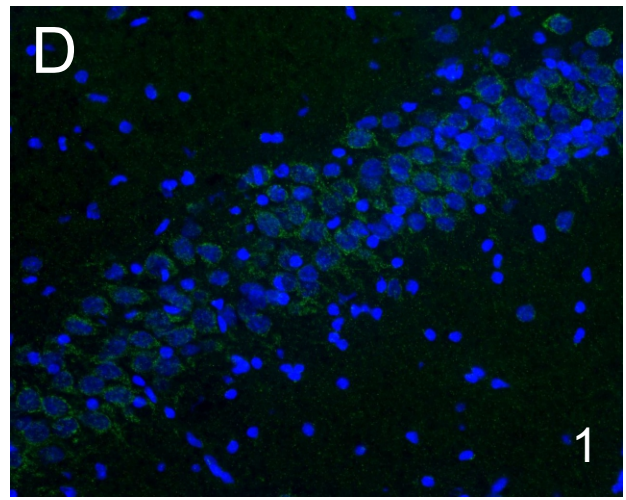

Hippocampus (CA1)

Supplement: Supplementary file 1 [file life-13-01826-s001.zip › Figure S2 - Hippocampal Bregma Diagram.pdf]
